# Supplementary material for: Bow-tie structures of twitter discursive communities
Source: Sci Rep. 2022 Jul 28;12:12944. doi: 10.1038/s41598-022-16603-7 (PMC9332050; doi:10.1038/s41598-022-16603-7)
Supplement: Supplementary file 1 — Supplementary Information. [file 41598_2022_16603_MOESM1_ESM.pdf]

Bow-Tie Structures of Twitter Discursive Communities
-Supplementary Material-

Mattia Mattei[1,2], Manuel Pratelli[1,3], Guido Caldarelli[4,5,1], Marinella Petrocchi[3,1], and Fabio Saracco[6,1,7,*]

[1]IMT School For Advanced Studies Lucca, p.zza San Francesco 19, 55100 Lucca, Italy
[2]Alephsys Lab, Universitat Rovira i Virgili, Av. Paisos Catalans 26 Tarragona, Tarragona E-43007, Catalonia, Spain
[3]Institute of Informatics and Telematics, National Research Council, via Moruzzi 1, 56124 Pisa, Italy
[4]Ca' Foscari University of Venice, Department of Molecular Sciences and Nanosystems, Ed. Alfa, Via Torino 155, 30170, Venezia Mestre, Italy
[5]European Centre for Living Technology (ECLT), Ca' Bottacin, 3911 Dorsoduro Calle Crosera, 30123 Venice, Italy
[6]Institute for Applied Mathematics "Mauro Picone", National Research Council, via dei Taurini 19, 00185 Roma, Italy
[7]Centro Ricerche "Enrico Fermi", via Panisperna 89 A, 00184 Roma, Italy
[*]Corresponding author: fabio.saracco@cref.it

ABSTRACT

This supplementary material contains all the information, the graphics and the statistics of the data sets not directly analysed in the main paper. In fact, for convenience, in the paper we presented only the results about the Italian Covid-19 dataset. Moreover, here we present a brief description of the algorithm computing and identifying the bow-tie structures in direct networks.

1 German Covid-19 dataset

The German Covid-19 dataset contains 1,552,106 tweets shared between February 2 and April 23, 2020. we identified the following discursive communities:

- **AfD**: this group contains accounts of politicians of the German nationalist and right-wing party "Alternative for Germany (AfD)";
- **LEFT-WING**: this community collects politicians of various German left-wing parties, as the "Social Democratic Party (SPD)", "Alliance 90/The Greens" and "Die Linke" (literally, "the left");
- **GOVERNMENT**: in this community are placed official accounts of German ministries and institutions as the Foreign, Defense or Health Ministries. It also contains politicians of the "Christian Democratic Union of Germany (CDU)";
- **MEDIA**: it includes the official accounts of the main German newspapers, blogs, TV-channels, journalists and other media in general.

Table 1 and the bar chart in Fig. 1 show the dimension of each discursive community. As for the other Covid-19 datasets, the MEDIA group results the most numerous one, with approximately 70% of the nodes of the entire network.
Fig. 2 shows the bow-tie structures for the four discursive communities. As in the main text, the dimension of the

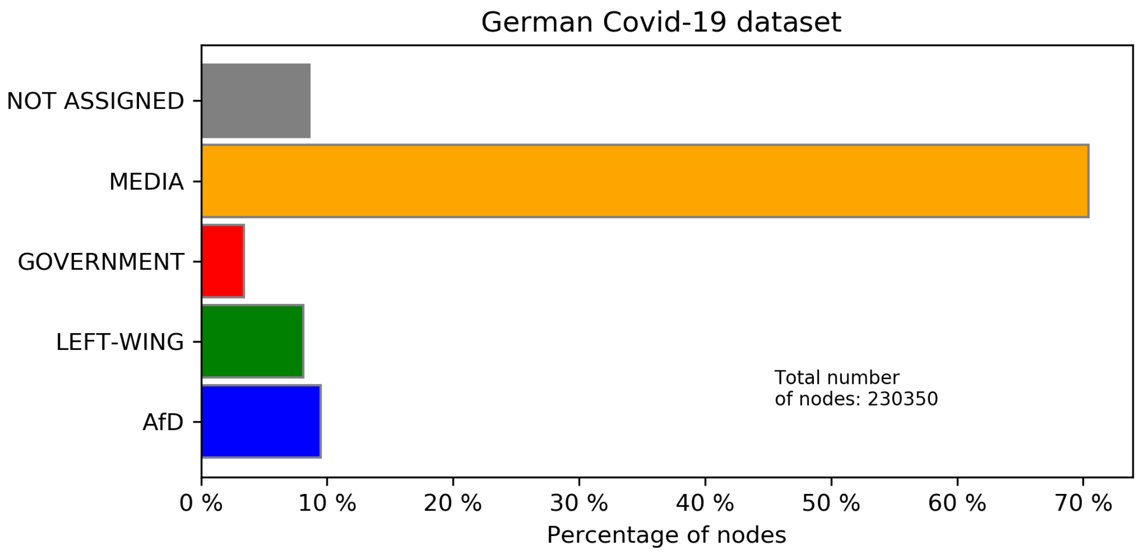

Figure 1. The dimension of the discursive communities of the German Covid-19 dataset. The bar chart displays the percentage of nodes in each discursive community. As for the other Covid-19 dataset the MEDIA group results the most numerous one, with approximately 70% of the nodes of the entire network.

COMMUNITY	DIM. (verified accounts)	DIM. (all accounts)
AfD	104	21903
L.-W.	161	18674
GOV.	79	7804
MEDIA	224	162215

Table 1. Dimension of the discursive communities of the German Covid-19 dataset, considering verified accounts only and all users.

COMMUNITY	DIM. (verified accounts)	DIM. (all accounts)
R.-W.	194	76243
L.-W.	228	85750
GOV.	397	65190
MEDIA	303	323280

Table 2. Dimension of the discursive communities of the French Covid-19 dataset, considering verified accounts only and all users.

sectors is proportional to the number of nodes and the color indicates the p-value encoding the mismatch with the predictions of the Direct Configuration Model (described in the Methods section of the main text). The AfD, GOVERNMENT and MEDIA groups display informative bow-tie structures; all of them are OUT-dominant, but only AfD has a strong bow-tie. In the LEFT-WING community the bow-tie is uninformative, with approximately 60% of the vertices in the OTHERS sector. In agreement with the results of the Italian Covid-19 dataset, the OTHERS block results significantly less numerous for the three communities with an informative bow-tie, and not for the LEFT-WING.

Also in this dataset, the AfD discursive community, which contains right-oriented and conservatives accounts, shows a more numerous and denser SCC. It is the only community with above 10% of the nodes and 25% of the links within the SCC, in which each vertex has over 20 links on average attached to it (see Fig. 3). The accounts in the AfD discursive community are those who retweets the most urls of web-pages indicated by Newsguard as untrustworthy. Indeed, we found approximately 3,500 retweets of this type in AfD, about 200 in MEDIA, 20 in LEFT-WING and even none in GOVERNMENT. For AfD, 30% of them originates from the SCC and ends in the OUT sector, 25% between IN and OUT and 20% remains in the SCC. Therefore, in over 50% of the cases an user shares untrustworthy contents from the SCC.

2 French Covid-19 dataset

The French Covid-19 dataset consists in 3,052,708 posts published between March 23 and April 7 about the epidemic. We identified 4 different discursive communities:

- **RIGHT-WING**: it collects conservatives and right-oriented accounts from French parties like “Rassemblement National”, “Les Républicains” and “Les Identitaires”;
- **LEFT-WING**: in this community there are the politicians and the supporters of center-left French parties like “La France Insoumise” or the socialists party (“Parti Socialiste”);
- **GOVERNMENT**: it collects accounts of institutions and ministries like the official account of the French government or that of “Ministère des solidarités et de la santé” (Ministry of Solidarity and Health). It also contains politicians from the party “La République En Marche”, whose leader is president Macron;
- **MEDIA**: this is the usual community containing official accounts of various media and journalists.

As for the other Covid-19 datasets, the MEDIA community results the most numerous one, with approximately 60% of the nodes of the network (Fig. 4). Tab. 2 shows the communities’ dimensions.

For this dataset we could not make the comparisons with the predictions of the entropy-based null-model because of the huge dimension of its discursive communities. For these groups, the computation time for generating a sample of the graph ensemble and analysing their bow-tie structure became too long. Therefore, in the following plots there will be no information about the p-values.

Fig. 5 shows that each discursive community displays an informative bow-tie structure. Remarkably, each of them are OUT-dominant ones, with no less of 40% of the nodes in every OUT sector. The mismatch in the number of nodes and links in the SCC between the right-wing community and the others still holds, but at much less extent, see Fig. 6. When matching Newsguard’s data with Twitter’s ones, we get results similar to the ones observed in the other datasets: we found 979 retweets with urls to untrustworthy web-pages in the right-wing

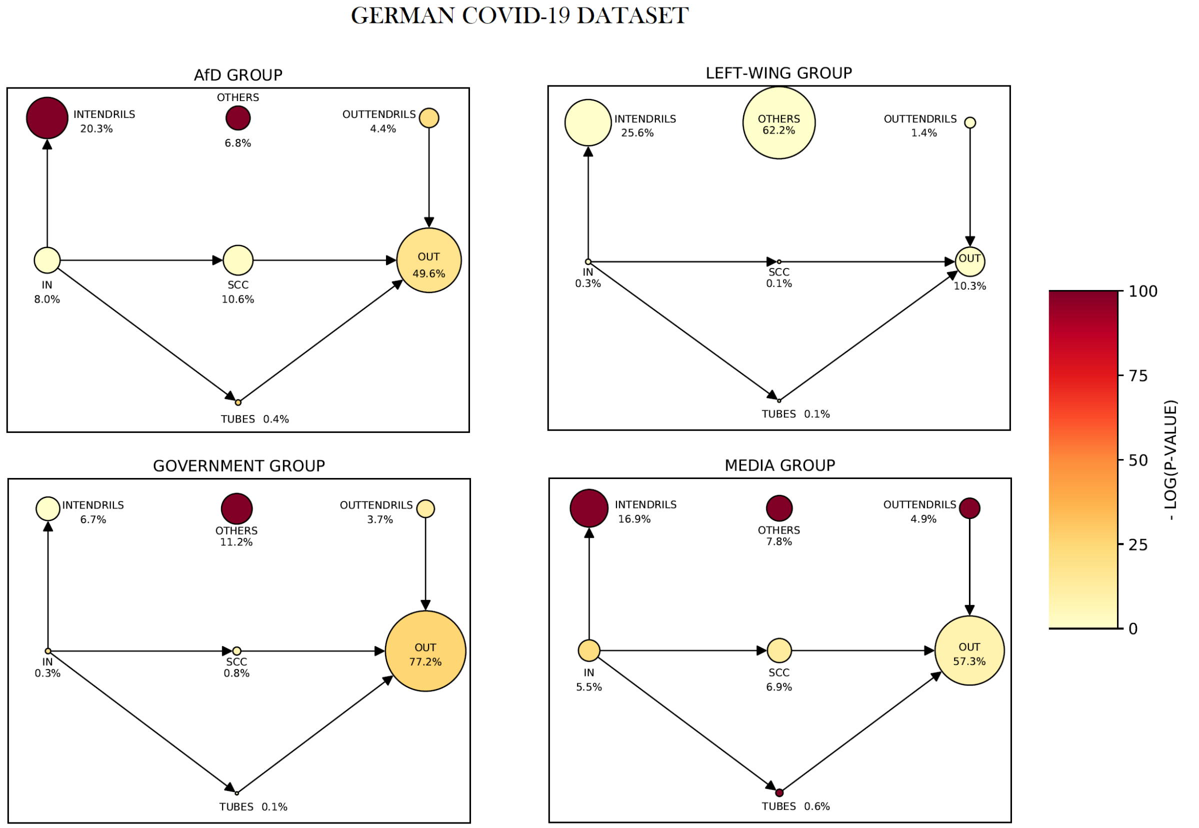

Figure 2. The bow-tie structure of the discursive communities of the German Covid-19 dataset. The dimension of the sectors is proportional to the number of nodes contained in them and the color indicates the distance between the observed and the predicted dimensions through $\ln(p - value)$. The AfD, Government and MEDIA groups display an informative bow-tie structure, i.e. the OTHERS sector is the represent less than 50% of the nodes. Considering the comparisons with the predictions of the Direct Configuration Model, the observed dimension for the OTHERS sector is significantly less numerous (considering a significance at 1%) for all the communities, apart for the LEFT-WING one.

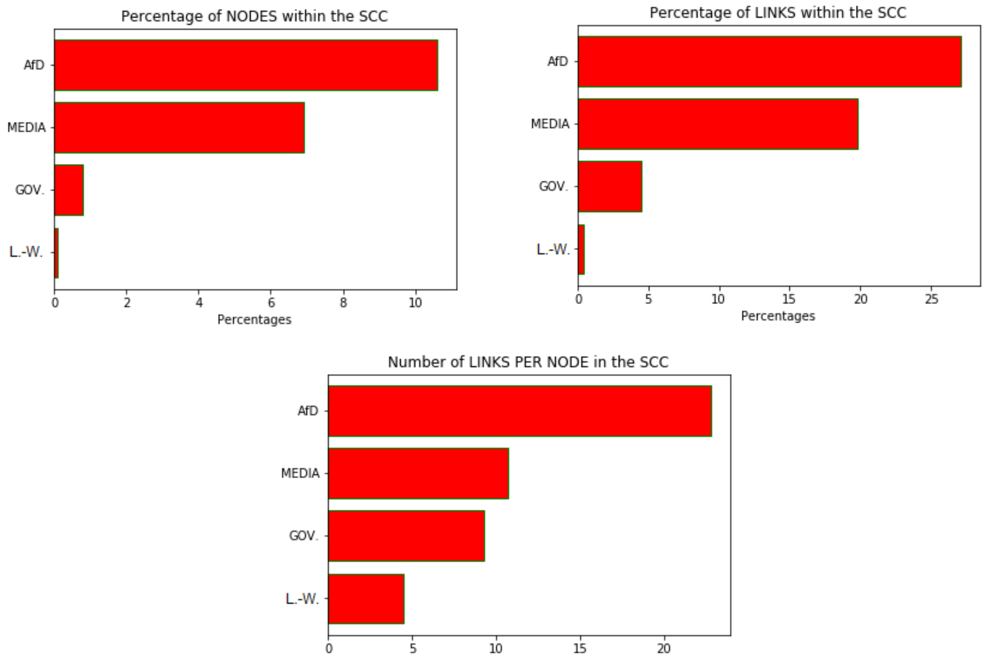

Figure 3. Percentage of nodes and edges in the SCC for the communities in the German Covid-19 dataset. As for the other datasets, in the German Covid-19 one the conservatives and right-oriented discursive community (AfD) has more numerous and denser SCCs, as it is displayed in the two top panels. In the bottom panel, it can be seen that also considering the fraction of links per node in the SCC, the AfD group results again the first one.

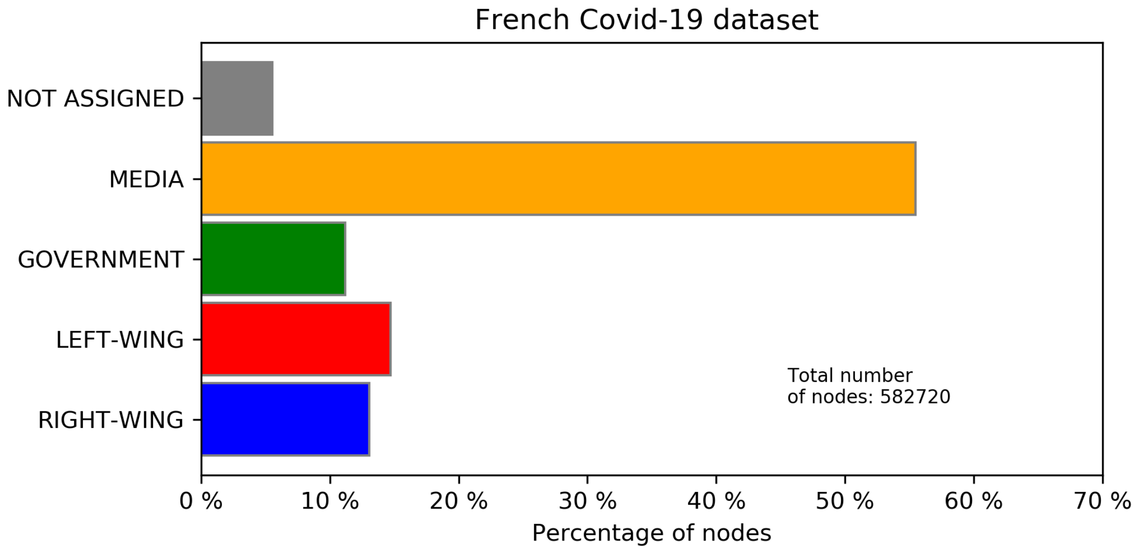

Figure 4. The dimension of the discursive communities of French Covid-19 dataset. The bar chart displays the percentage of nodes in each discursive community. The MEDIA group results the most numerous one, with approximately 60% of the nodes, as it happens in the other Covid-19 datasets.

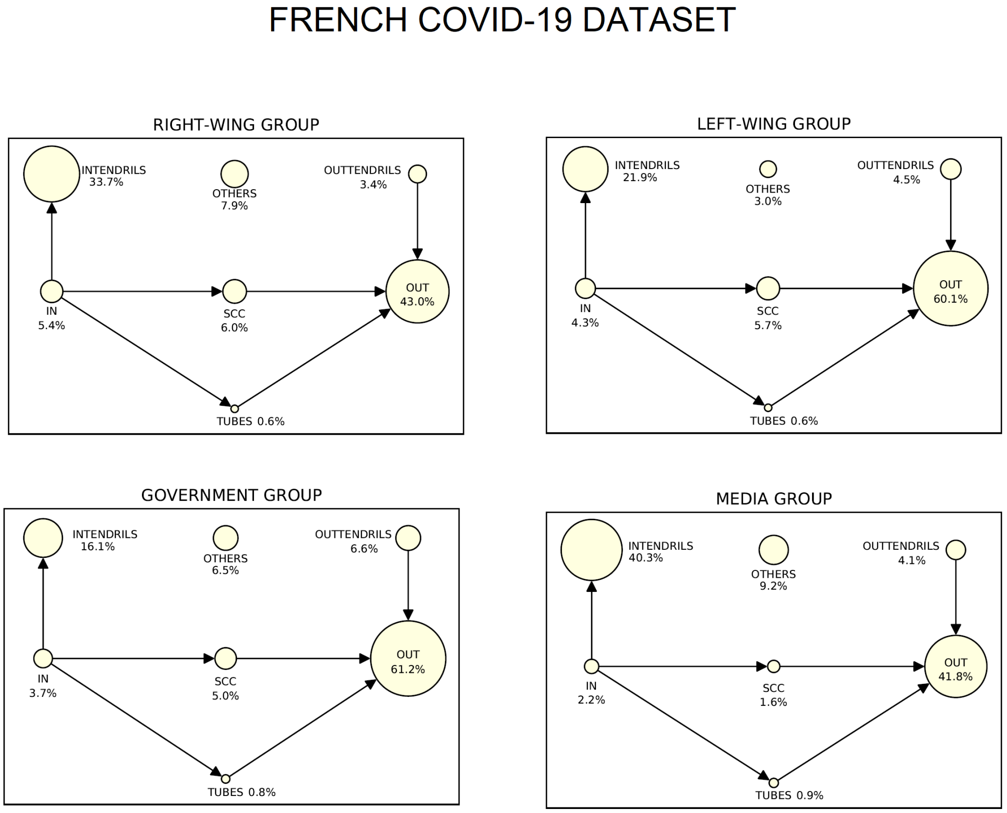

Figure 5. The bow-tie structure of the discursive communities of the French Covid-19 dataset. All discursive communities have informative bow-tie structures and all of them are OUT-dominant.

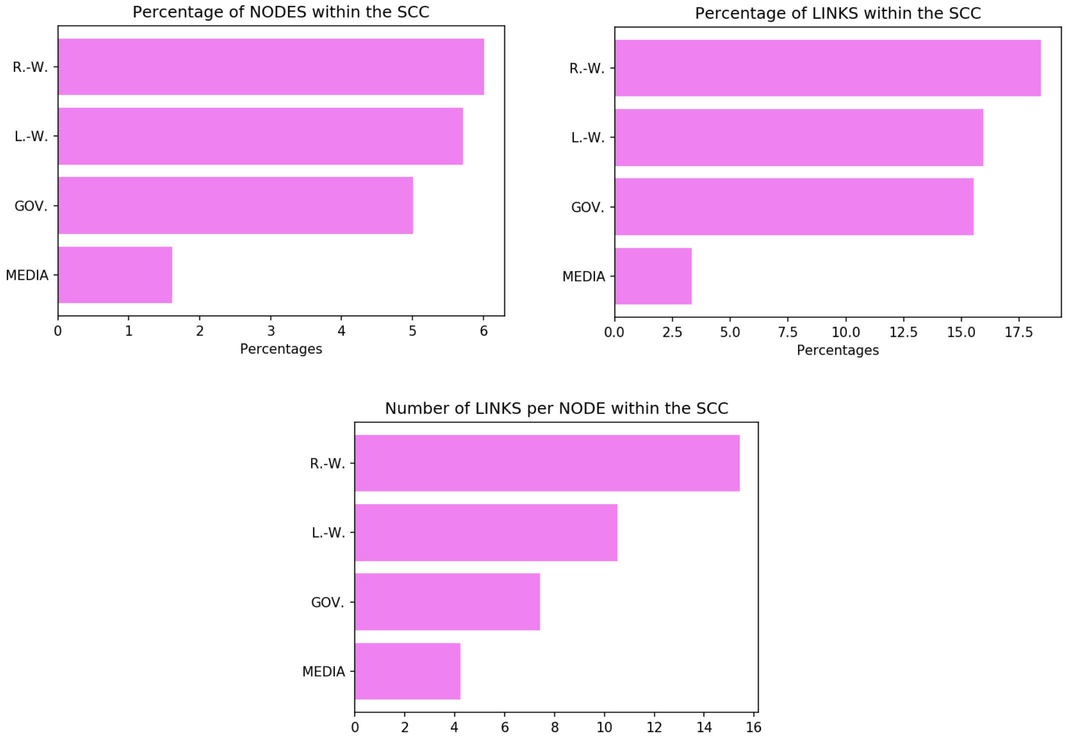

Figure 6. Percentage of nodes and edges in the SCC for the communities in the French Covid-19 dataset. The right-wing community has more links and nodes in the SCC, even if this time in much less extent respect to the other datasets.

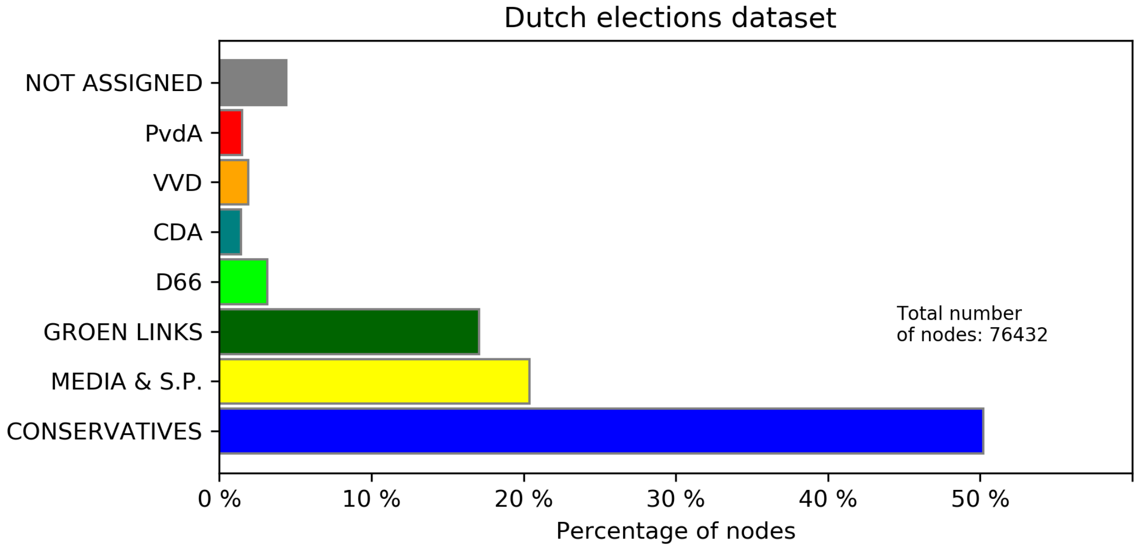

Figure 7. The dimension of the discursive communities of the Dutch elections dataset.
The bar chart displays the percentage of nodes in each discursive community. The CONSERVATIVES group results by far the most numerous one, with approximately 50% of the nodes of the entire network.

COMMUNITY	DIM. (verified accounts)	DIM. (all accounts)
CONS.	41	38366
MEDIA & S.P.	39	15570
GROEN LINKS	36	13057
D66	31	2409
CDA	18	1081
VVD	15	1453
PvdA	11	1133

Table 3. Dimension of the discursive communities of the Dutch Elections dataset, before and after the label propagation procedure (i.e., considering verified accounts only and all users).

community, 103 in the left-wing and none in the others. For the former community 25% are located between SCC and OUT, 22% between IN and OUT, 14% in the SCC, 12% between IN and SCC and much less between the other sectors. In the case of the left wing, 45% of these retweets are located between SCC and OUT and 31% in the SCC.

3 Dutch elections dataset

The Dutch elections dataset consists in 1,002,499 tweets posted between February 2 and March 31 2021. In this case almost each discursive community has the name of a specific Dutch politcal party: "GroenLinks" (center-left, green), "Christian Democratic Appeal (CDA)" (center, Christian-democratic), "Democrats 66 (D66)" (center/center-left, liberal), "People's Party for Freedom and Democracy (VVD)" (conservative-liberal) and "Labour Party (PvdA)" (center-left, social-democratic). Then we have the CONSERVATIVES community which collects accounts from right-oriented parties like "Party for Freedom" or "Forum for Democracy" and the MEDIA & S.P., which is the usual MEDIA community with a couple of accounts belonging to the Dutch "Socialist Party". Fig. 7 and Table 3 show the dimension of these seven discursive communities. As it is possible to observe in Fig. 8, all the discursive communities show an informative bow-tie structure, with the only exception of VVD.

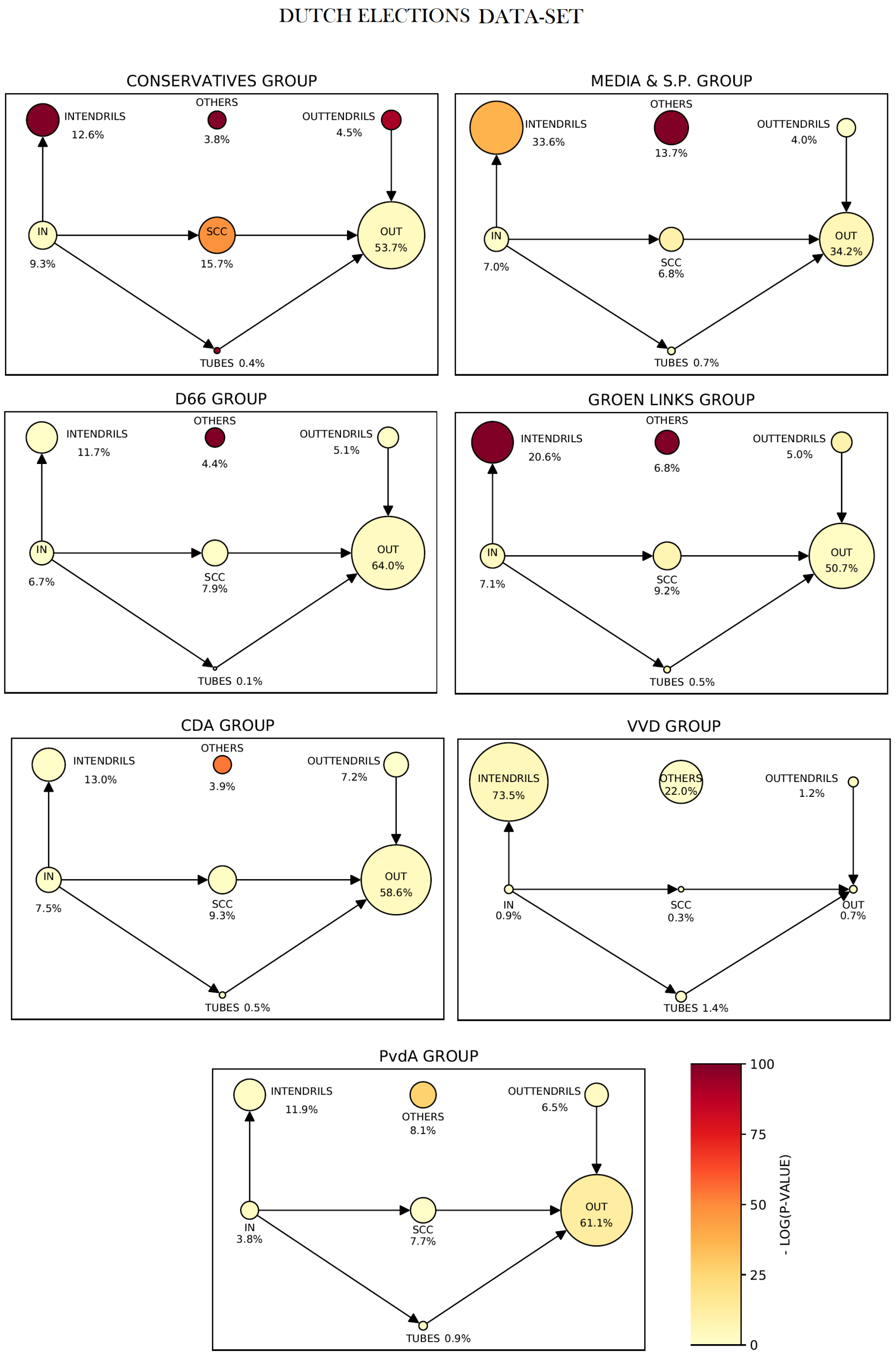

Figure 8. **The bow-tie structure of the discursive communities of the Dutch elections dataset.** All the discursive communities in this dataset display a strong bow-tie structure, but the VVD one.

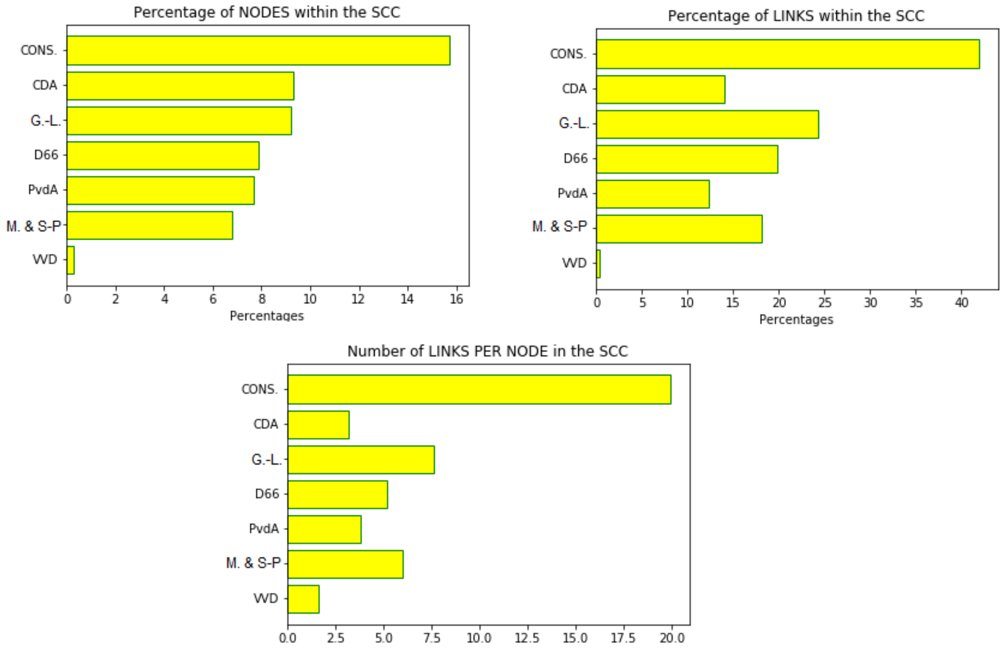

Figure 9. Percentage of nodes and edges in the SCC for the communities in the Dutch elections dataset. The conservative and right-oriented discursive community (CONS.) has more numerous and denser SCCs, as it is displayed in the two top panels. In the bottom panel, it can be seen that also considering the fraction of links per node in the SCC, the CONS. group outperforms other communties.

The CONSERVATIVES group results again that community with the highest percentages of nodes and links within SCC (see Fig. 9). Indeed, SCC contains more than 40% of the links of the entire network.
In this dataset are present just a few retweets containing urls to untrustworthy web-pages (Newsguard). However, they are all located in the CONSERVATIVES community: 153 in total, whose 55% in the SCC and 45% between SCC and OUT.

4 Italian debate on migrants dataset

This Italian dataset contains Twitter posts about the migration flows from Northern Africa. The dataset consists of 1,081,780 posts published between January 23, 2019 and February 22, 2019.
Using Louvain communitiy detection algorithm (see the main text), the network was partitioned in the following communities: DX (right-oriented Italian parties as “Lega Nord”), CSX (left-oriented Italian parties as the Democratic Party and other minor center-left parties), M5S (“Five Star Movement” party) and the usual MEDIA community. The first two communities result the most numerous ones (see Fig. 10 and Table 4).
In Fig. 11 there are the bow-tie structures for the four discursive communities in this dataset. The most numerous community of DX and CSX display informative bow-ties while in the two smaller ones the nodes are mostly located in the OTHERS sector, especially for the MEDIA community (above 95%). Looking to the colors in the graphs, in general, the latter ones result more in agreement with the Direct Configuration Model.

The DX community, which again contains politicians of right-oriented Italian parties, has the most numerous and denser SCC (Fig. 12), such that on average a node therein has over 25 links.
Newsguard data tell us that 15,160 retweets in the DX network contain the urls of untrustworthy web-pages, while only 14 for the CSX, 3 for MEDIA and none for M5S. In the case of the DX community, 59% of them can be found inside the SCC and 36% between the SCC and OUT.

COMMUNITY	DIM. (verified accounts)	DIM. (all accounts)
DX	17	34235
CSX	51	45940
M5S	14	396
MEDIA	25	1293

Table 4. Dimension of the discursive communities in the dataset about the Italian debate on migrants, considering verified accounts only and all accounts.

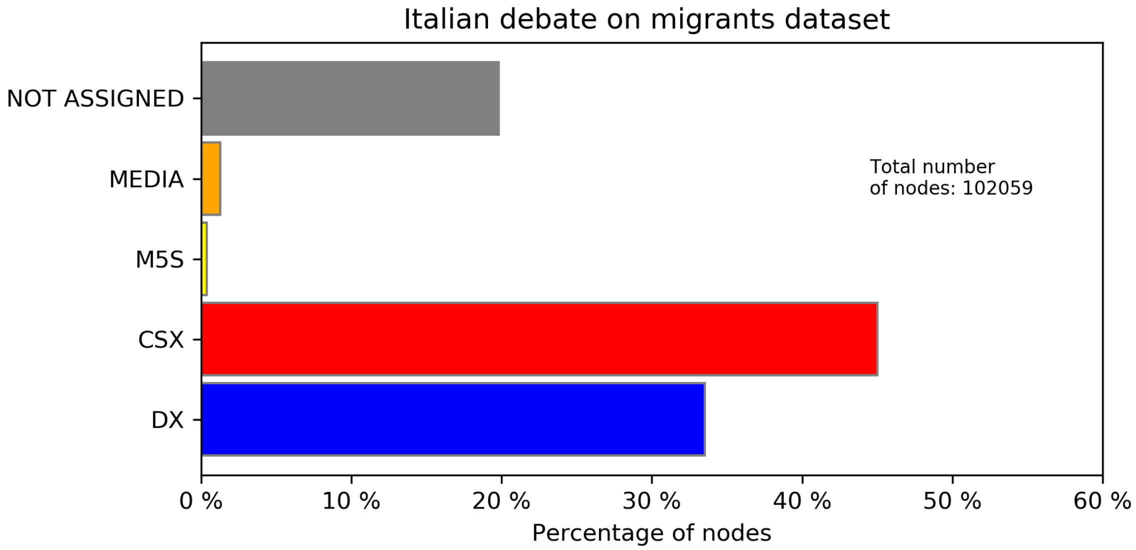

Figure 10. The dimension of the discursive communities of Italian debate on migrants dataset. The bar chart displays the percentage of nodes in each discursive community. The DX and CSX groups result the most numerous ones, with between 30% and 50% of the nodes. Among the ones we analysed, this is the only dataset in which the percentage of nodes not assigned to a discursive community by the label propagation procedure overcome 15%.

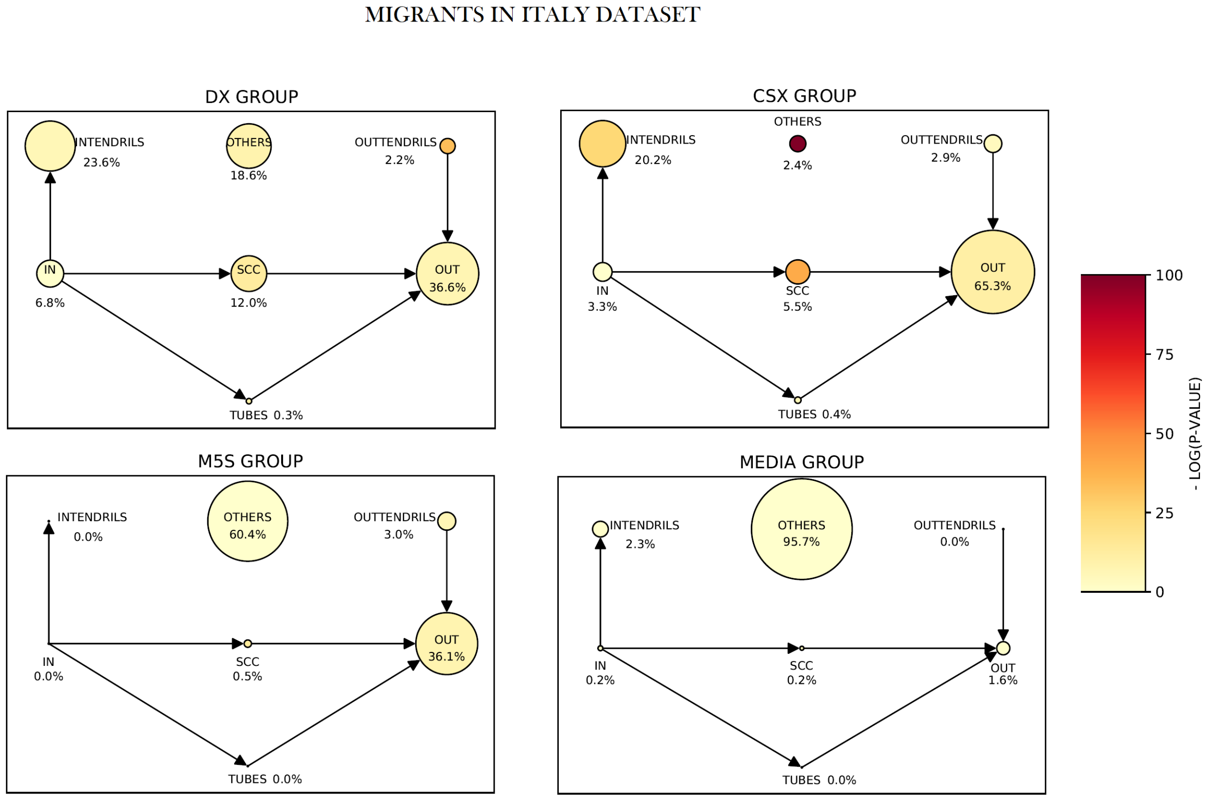

Figure 11. The bow-tie structure of the discursive communities of the Italian debate on migrants dataset.
The most numerous community of DX and CSX display informative bow-ties (only the CSX one is strong), while in the smaller ones of M5S and MEDIA the nodes are mostly located in the OTHERS sector. Looking to the colors in the graphs, M5S and MEDIA are more in agreement with the predictions of the DCM.

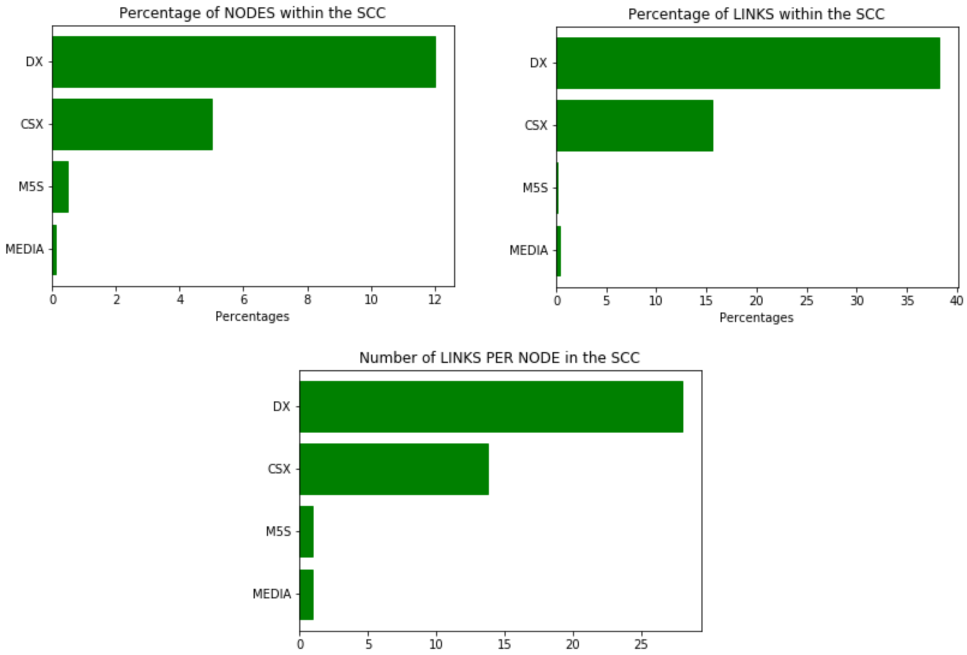

Figure 12. Percentage of nodes and edges in the SCC for the communities in the Italian debate on migrants dataset.
Again, the conservative and right-oriented discursive community (DX) has more numerous and denser SCCs, as it is displayed in the two top panels. In the bottom panel, it can be seen that also considering the fraction of links per node in the SCC, the DX group results again the first one.

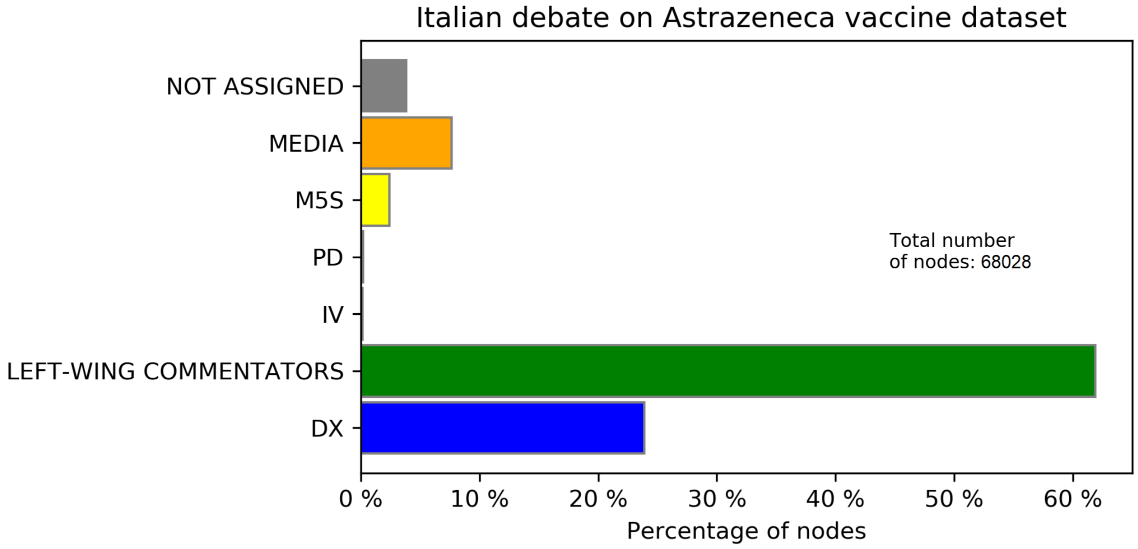

Figure 13. The dimension of the discursive communities of Italian debate on Astrazeneca vaccine dataset.
In this bar chart the percentage of nodes in each discursive community is displayed. All communities but MEDIA and PD display informative bow-ties, and, among them, the DX and IV ones are strong. The LEFT-WING COMMENTATORS group results the most numerous one, with over 60% of the nodes.

5 Italian debate on Astrazeneca vaccine

This dataset contains 583,236 Twitter posts published in Italy and regarding the discussion about the safety of Astrazeneca vaccine against Covid-19. The dataset contains posts shared between March 15, 2021 and May 15, 2021. we identified the following discursive communities:

- **DX**: this is the usual right-oriented and conservatives community found even in the other Italian datasets, i.e. it contains accounts from the "Lega" and "Fratelli d'Italia" parties;
- **PD**: the Italian Democratic Party (center-left);
- **IV**: it collects the politicians of the "Italia Viva" party (center-left);
- **LEFT-WING COMMENTATORS**: this particular community is formed by several well-known personalities, often left-oriented, which are not politicians but journalists, blogger, actors or entertainers. This community contains also the most famous Italian epidemiologist Roberto Burioni;
- **M5S**: the Italian populist party "Movimento 5 Stelle";
- **MEDIA**: the usual community containing official accounts of newspaper, blog, TV-channels, radio and others.

The distribution of the nodes in these six communities is showed in Fig. 13 and the related dimensions are shown in Table 5. The two biggest communities, DX and LEFT-WING COMMENTATORS, show a respectively strong and weak bow-tie structures (Fig. 14), denoting, once more, that the strength of the structure does not depend on its dimension. Nevertheless, thery are both OUT-dominant. In the M5S and IV ones there is a nearly balanced situation between INTENDRILS and OUT as the dominant sector. While MEDIA bow-tie is poorly informative, the PD community is not informative, with over 50% of the nodes in the OTHERS.

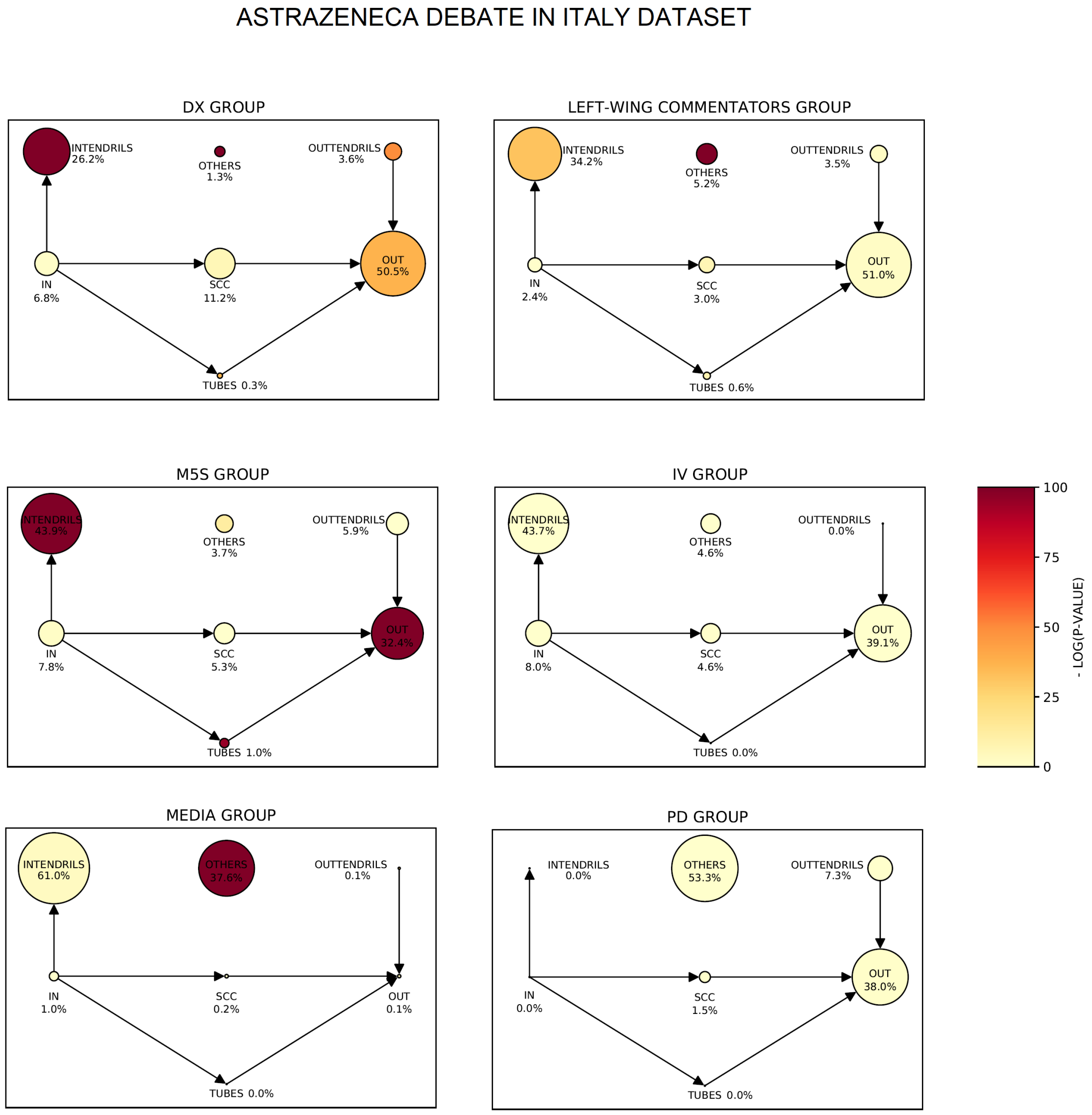

Figure 14. The bow-tie structure of the discursive communities of the Italian debate on Astrazeneca vaccine dataset.
The DX and LEFT-WING COMMENTATORS communities display informative bow-tie structure; nevertheless they are both OUT-dominant ones. In the weak bow-ties of M5S and IV there is a balanced situation between INTENDRILS and OUT as the dominant sector. The PD community does not display an informative bow-tie structure, with over 50% of the nodes in the OTHERS.

COMMUNITY	DIM. (verified accounts)	DIM. (all accounts)
DX	22	16249
L.-W. COMM.	17	42112
M5S	19	1635
MEDIA	34	5197
IV	11	88
PD	12	140

Table 5. Dimension of the discursive communities in the dataset on the Italian debate on Astrazeneca vaccine, before and after the label propagation procedure (i.e., considering verified accounts only or all accounts).

The DX community results again the community with the most numerous and denser strongly connected component; in DX community, the average degree of nodes in SCC is greater than 27 links, while in the other communities there are always less than 10 links per node (Fig. 15). According to Newsguard, 728 retweets in DX community contained urls to untrustworthy pages. They are distributed as follow: 43% between SCC and OUT, 26% in the SCC, 13% between IN and SCC, 12% between IN and OUT and much less between the other sectors. We found only two retweets of this type in the M5S community and none in the others.

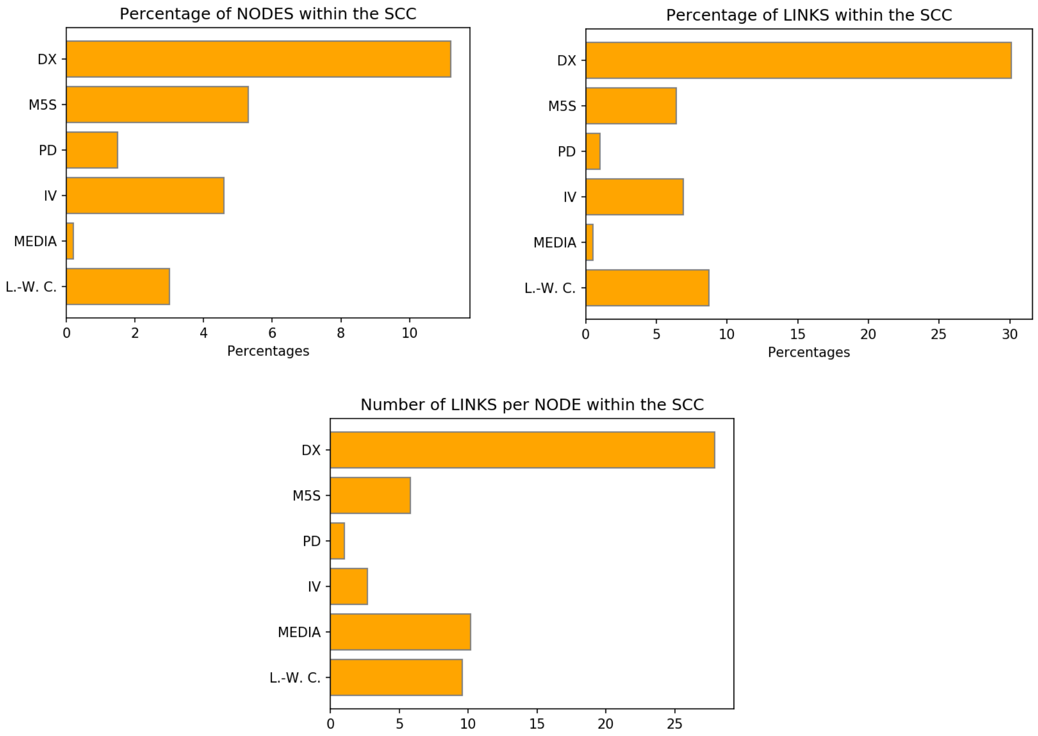

Figure 15. Percentage of nodes and edges in the SCC for the communities in the Italian debate on Astrazeneca vaccine dataset.
Again, the conservative and right-oriented discursive community (DX) has more numerous and denser SCCs, as it is displayed in the two top panels. In the bottom panel, it can be seen that also considering the fraction of links per node in the SCC, the DX group results again the first one.
